# Inequality in the distribution of resources in the health sector before and after the Health Transformation Plan in Qazvin, Iran

**DOI:** 10.1186/s41043-023-00495-y

**Published:** 2024-01-02

**Authors:** Asghar Nasiri, Mohammad Amerzadeh, Hasan Yusefzadeh, Saeideh Moosavi, Rohollah Kalhor

**Affiliations:** 1 Student Research Committee, School of Public Health, Qazvin University of Medical Sciences, Qazvin, Iran; 2 Social Determinants of Health Research Center, Research Institute for Prevention of Non-Communicable Diseases, Qazvin University of Medical Sciences, Qazvin, Iran; 3 Department of Health Management and Economics, School of Public Health, Urmia University of Medical Sciences, Urmia, Iran

**Keywords:** Health resources, Gini coefficient, Hirschman–Herfindahl index, HTP

## Abstract

**Background:**

The equitable distribution of healthcare resources represents a paramount objective in the realm of global health systems. Thus, the present study sought to assess the fairness in the allocation of health resources at Qazvin University of Medical Sciences (QUMS), both prior to and subsequent to the implementation of the Health Transformation Plan (HTP) using the Gini coefficient and the Hirschman–Herfindahl index (HHI).

**Methods:**

This descriptive-analytical study aimed to investigate the distribution of healthcare resources among general practitioners (GPs), specialists, and subspecialists employed at QUMS between 2011 and 2017. Demographic data pertaining to the cities were obtained from the statistical yearbooks of the Statistical Center of Iran, while information regarding the healthcare workforce was extracted from QUMS records. The analysis utilized two key measures, namely the Gini coefficient and the HHI, to assess the fairness of resource distribution. Data analysis was performed using Microsoft Excel 2016 and the Stata statistical software.

**Results:**

The highest number of GPs, specialists, and subspecialists was observed in 2014, 2017, and 2017, respectively, while the lowest number was recorded in 2016, 2011, and 2015, respectively. From 2011 to 2017, the Gini coefficient for GPs ranged between 0.61 and 0.63. Among specialists, the lowest Gini coefficient value was observed in 2015 (0.57), while the highest was recorded in 2017 (0.60). The Gini coefficient for subspecialists remained constant at 0.52 from 2011 to 2017. The HHI revealed a high concentration of GPs in the cities of Qazvin province. Although the disparity gradually decreased in the specialties of eye, ear, nose, and throat, and pediatrics, the concentration still persists in Qazvin. In general surgery, the index value is low, indicating some level of inequality. In anesthesia and neurology, the index value decreased after the HTP and reached 5700; however, achieving equality (below 1000) still requires further efforts. No significant change in the index was observed after the HTP in specialties such as neurosurgery, rehabilitation, and nuclear medicine. Subspecialists also exhibited a concentration in the city of Qazvin.

**Conclusion:**

Based on the analysis of the Gini and HHI, it is evident that the distribution of GPs has not undergone significant changes following the implementation of the HTP. The Gini coefficient, which ranges from 0.4 to 0.6, indicates a high to complete level of inequality in the distribution of specialists and subspecialists. Moreover, the HHI exceeds 1000, reflecting a concentration of resources in specific areas. As a result, the HTP has not yet achieved its goal of ensuring a fair distribution of human resources. To address this issue, it is recommended to redesign distribution policies, including the allocation of physical health resources, such as specialized hospitals, beds, and medical equipment. Additionally, increasing student admissions in specialized and subspecialized fields and implementing tariff incentives can contribute to a more equitable distribution of resources. By aligning distribution policies with the principle of fairness, the healthcare system can better address the issue of resource distribution.

## Background

Ensuring equitable access to healthcare is a fundamental principle of modern democracies, as it promotes equal opportunities for individuals to receive necessary medical services [1]. The effective distribution of human resources is vital in achieving this goal, not only in developed nations such as Finland but also in developing countries that aim to improve healthcare equity. However, the Commission on Social Determinants of Health has identified significant disparities in health outcomes among countries, often stemming from inequitable distribution of healthcare resources. Such disparities can lead to inefficient utilization of resources and impose additional financial burdens on patients [2, 3].

The unequal distribution of healthcare professionals, particularly doctors, has emerged as a growing concern in the global healthcare sector [4]. For example, in Japan, Matsumoto et al. found a significant increase in the Gini coefficient after 2006, indicating an inappropriate distribution of doctors [5]. Moreover, the inequality ratio in the allocation of specialist services exceeds that of general practitioners (GPs) [5]. In Iran's West Azerbaijan province, Mustafavi et al. identified an unfair distribution of specialists [6]. Similarly, Maskarpour Amiri et al. examined the distribution of intensive care unit (ICU) beds in Iran and observed an increase in inequality, as evidenced by the rising Gini coefficient between 2010 and 2012 [7].

In 2013, the Ministry of Health and Medical Education (MoHME) in Iran implemented a significant healthcare system transformation known as the Health Transformation Plan (HTP). The HTP included eight service packages, several of which were designed to promote fairness and improve access to healthcare resources. One of the goals of the HTP was to enhance physician retention in underserved areas, with the potential to impact the distribution of physicians across different regions [8, 9].

The evaluation of the HTP requires a comprehensive assessment of its outcomes, particularly in relation to health equity. Measuring health equity poses a significant challenge, leading different nations to adopt varied methodologies. Experts in statistics and economics have proposed different approaches to evaluate the fairness of service distribution. Among these methods, the Gini coefficient is considered a suitable measure as it provides a balanced ratio that is independent of average measurements [4]. Additionally, the Hirschman–Herfindahl index (HHI) is significant as one of the indices used to quantify the concentration of market resources. This index is valuable as it considers all points along the concentration curve, utilizing the available information throughout the curve. Therefore, it serves as a useful index for assessing both concentration and inequality in resource allocation [10].

This study aims to evaluate the distribution of GPs, specialists, and subspecialists within QUMS, Iran, both before and after the HTP in 2014. The HTP is designed to promote justice and equitable access to healthcare resources. In order to assess the impact of the HTP, this study utilizes two key measures: the Gini coefficient and the HHI. These quantitative tools are employed to analyze the distribution patterns of healthcare professionals and determine any changes that may have occurred as a result of the implementation of the HTP.

## Methods

This study takes a descriptive and analytical approach to evaluate the equity in the distribution of health resources at QUMS before and after the implementation of the HTP. The assessment is conducted by utilizing two key indices: the Gini coefficient and the HHI.

The study population consists of subspecialists, specialists, and GPs affiliated with QUMS between 2011 and 2017. Data collection involved gathering information on the number of doctors practicing within healthcare networks in each city during the specified time period. As national census data on city and province populations are conducted every five years, population statistics for the target cities in 2010 and 2015 were obtained from statistical yearbooks. To estimate the province populations for the respective years, a combined method from the Iran Statistics Center was employed. To ensure data accuracy, cross-referencing and verification were conducted by accessing demographic information available from the Deputy for Public Health at QUMS. The collected data were then validated using Excel and Stata 2016 software, and subsequently, the Gini coefficient and HHI were calculated to assess the distribution of health resources.

The Gini coefficient, developed by Italian statistician Gini, is a quantitative measure used to assess income distribution. It ranges from zero to one, where zero represents complete equality and one represents complete inequality [11]. A Gini coefficient value below 0.2 indicates a state of complete equality in the distribution, while a coefficient between 0.2 and 0.3 suggests a significant degree of equality. A range of 0.3–0.4 implies inequality in the distribution, and values between 0.4 and 0.6 represent varying degrees of inequality. Gini coefficients exceeding 0.6 indicate a state of absolute inequality [11].

The formula for calculating the Gini coefficient, as proposed by Brown, is as follows:

$$G=1-\sum_{i=0}^{K-1}({Y}_{i+1}+{Y}_{i})({X}_{i+1}-{X}_{i})$$

In this formula, ‘*X*’ represents the relative cumulative frequency of the population, and ‘*Y*’ stands for the relative cumulative frequency of the physician [11].

After computing the Gini coefficient, the study further utilized the HHI to examine concentration within the health system. The HHI is a measure used to evaluate the concentration and share of companies within related industries. It is calculated by summing the squared power of each company's share in the total production of the industry [12]. In the specific context of this study, the HHI was utilized to assess the level of concentration in the distribution of GPs, specialists, and subspecialists across various cities within Qazvin province [13]. The formula for calculating the Hirschman–Herfindahl index, considering the collected data, involves the following variables: *n*, representing the number of cities in Qazvin province, and *S*, denoting the share of GPs, specialists, and subspecialists in each city. The share is determined using the formula:

$${\text{HI}} = \sum\limits_{i = 1}^{n} {S_{i}^{2} } \qquad {\text{HI}} = \sum\limits_{i = 1}^{n} {\left( {\frac{{X_{i} }}{X}} \right)^{2} }$$

X_i_ variable number by city, X is the number of the same variable in the whole province.

## Results

Table 1 displays data regarding the number of GPs, specialists, and subspecialists in QUMS. The table indicates that the allocation of GPs has demonstrated a gradual increase with the implementation of the HTP, followed by a slight decrease over time. On the other hand, there has been a notable and significant rise in the number of specialists, particularly in the field of subspecialties.

**Table 1:** Distribution of GPs, specialists, and subspecialists

Year	GPs	Specialists	Subspecialists
2011	542	289	29
2012	534	297	28
2013	545	318	29
2014	559	311	28
2015	521	301	28
2016	521	397	39
2017	395	456	64

Gini coefficient of GPs, specialists and subspecialists

The highest Gini coefficient value was observed among cardiologists, indicating a state of complete inequality in the distribution of this specialty. Similarly, after the HTP, the category of emergency medicine specialists exhibited the highest Gini coefficient, also suggesting a state of complete inequality. For other fields, the Gini coefficient values ranged between 0.4 and 0.6, indicating a substantial degree of inequality in the distribution of GPs across different years. Table 2 provides a detailed overview of the Gini coefficient values specifically for GPs across different years. This table presents information on the level of inequality in the distribution of GPs within the studied population during each specified year.

**Table 2:** Gini coefficient of GPs by year

Year	2011	2012	2013	2014	2015	2016	2017
Gini	0/62	0/61	0/62	0/62	0/63	0/62	0/64

The data analysis consistently shows that the Gini coefficient index for the distribution of GPs remains above 0.6 throughout the entire period under study. This implies a high level of inequality in the distribution of GPs. Upon conducting a trend analysis, it becomes evident that the implementation of the HTP did not lead to a reduction in the Gini coefficient index or promote equality in the distribution of GPs.

Based on the data presented in Table 3, the Gini coefficient values for the years under consideration fall within the range of 0.5 to 0.6. These values indicate a high level of inequality in the distribution of GPs, specialists, and subspecialists. Notably, the changes in the Gini coefficient between 2013 and 2017 demonstrate the most significant levels of inequality, suggesting a state of absolute inequality during those years. This indicates a highly uneven distribution of healthcare professionals within the studied population. However, it is worth mentioning that the implementation of the HTP had some impact on reducing inequality between 2014 and 2016. During this period, the Gini coefficient exhibited a decreasing trend, indicating a reduction in inequality.

**Table 3:** Gini coefficient of all specialists

Year	2011	2012	2013	2014	2015	2016	2017
Gini	0/59	0/59	0/60	0/58	0/57	0/59	0/60

Based on the data presented in Table 4, the Gini coefficient values for the specified period from 2011 to 2017 did not show significant changes. The trend analysis indicates that the Gini coefficient consistently remains within a range that indicates a high level of inequality in the distribution of GPs, specialists, and subspecialists.

**Table 4:** Total Gini coefficient for subspecialists

Year	2011	2012	2013	2014	2015	2016	2017
Gini	0/53	0/52	0/52	0/52	0/52	0/52	0/52

### HHI of GPs, specialists, and subspecialists

The HHI a measure used to quantify the concentration of health resources across different cities. It is calculated by summing the squares of each city's share of these resources. A lower index value, typically below 1000, indicates a lower concentration of resources within the cities, suggesting a more equitable distribution. In cases where a particular field or resource is absent in a specific city, a zero value is assigned in the corresponding table entry to indicate the absence of that field in that particular city. This allows for a clear representation of the distribution and concentration of health resources across the different cities being studied.

### HHI of GPs by year

Based on the information presented in Table 5, it is evident that there is a high concentration of GPs in Qazvin province, indicating an uneven distribution of GPs across the different regions within the province. This highlights the need for a more balanced and equitable distribution of GPs across various areas of the province. Additionally, the table suggests that there has been some improvement in the inequality of GP distribution over the specified period, particularly after the implementation of the HTP. The index value, which represents the degree of inequality, has gradually decreased over time, indicating a reduction in the concentration of GPs within certain regions. However, it is important to note that there was an exception in 2016, where the index value did not show a decline.

**Table 5:** Hirschman–Herfindahl index of GPs in Qazvin province by year

City	Year						
	2011	2012	2013	2014	2015	2016	2017
Hirschman index	5108/87	5445/33	5028/47	4925/39	4694/08	5227/41	4346/23

### HHI of specialists by year

Based on the information presented in Table 6, it is evident that there is a high concentration of specialties within Qazvin province, indicating an imbalanced distribution across different regions, particularly concerning the population of cities. This highlights the need for a more equitable allocation of specialties across the province. The table also indicates that inequality in the distribution of specialties has varied across different fields. In particular, inequality in the distribution of specialties such as emergency medicine, radiology, ENT, ophthalmology, dermatology, and psychiatry has increased compared to the years prior to the implementation of the HTP.

**Table 6:** Hirschman–Herfindahl index of specialists in Qazvin province by year

City	Year						
	2011	2012	2013	2014	2015	2016	2017
Hirschman index	6233/11	6283/93	5893/28	6762/30	6968/12	6152/94	6466/87

The distribution in some specialties, including rehabilitation, nuclear medicine, oncology, forensics, social medicine, orthopedics, infectious diseases, neurosurgery, radiotherapy, and traditional medicine, has not varied. It is high in comparison to the years prior to the HTP.

Based on the information presented in Table 7, it is evident that there is a high concentration of subspecialties within Qazvin province, indicating an unbalanced and unequal distribution across different regions. The table further indicates that, even after the implementation of the HTP, some fields continue to have an unbalanced distribution and high concentration of subspecialties. This suggests that the HTP may not have effectively addressed the issue of unequal distribution in these particular fields.

**Table 7:** Hirschman–Herfindahl index of subspecialists in Qazvin province

City	Year						
	2011	2012	2013	2014	2015	2016	2017
Hirschman index	9334/12	10,000	10,000	10,000	10,000	10,000	10,000

## Discussion

The study aimed to assess the equality in the distribution of health resources, including GPs, specialists, and subspecialists, in Qazvin province before and after the implementation of the HTP. The analysis utilized the Gini coefficient and the HHI to evaluate resource distribution. The findings of the study indicate that the HTP did not effectively address the issue of equitable resource distribution within the health sector of Qazvin province. The calculated Gini coefficient values for all health resources were high, indicating a significant level of inequality in their distribution. This suggests that there is an uneven allocation of health resources, including GPs, specialists, and subspecialists, among different regions within the province. Furthermore, the results obtained from the HHI further highlight the concentration and disproportionate distribution of human resources within the health sector of Qazvin province. This implies that certain areas or cities may have a higher concentration of health resources, leading to disparities in access to healthcare services.

The results indicate that both before and after the implementation of the HTP, the Gini coefficient for GPs in Qazvin province exceeded 0.6, indicating a high level of absolute inequality in the distribution of GPs. This finding is significant because GPs play a crucial role in providing primary healthcare services and are often the first point of contact for individuals seeking medical assistance. As primary healthcare is essential for promoting overall health and well-being, it is imperative to prioritize and closely examine the geographical distribution of GPs [14].

Haro et al.'s study in the USA revealed that the distribution of GPs has exhibited an increasing level of inequity and disparity across diverse regions [15]. Similar findings were observed in studies conducted in Greece and Albania, where the estimation of Gini coefficients for GPs pointed to varying degrees of inequality, underscoring the necessity for additional GPs to mitigate this imbalance [16]. Conversely, investigations concerning the geographical distribution of GPs in Japan and the United Kingdom yielded Gini coefficient values of 0.17 and 0.08, respectively, indicating a relatively lower level of inequality in comparison [17]. Rezaei and Zandian's study in Iran further confirmed the existence of disparities in the distribution of GPs [18]. However, Qadri's study conducted in Sistan and Baluchistan province reported a decrease in inequality, contradicting the findings of the present study [19].

The findings of the study revealed that the highest value of the Gini coefficient for specialists before the implementation of the HTP was associated with cardiologists. After the HTP, the highest value was linked to emergency medicine specialists. These results indicate complete inequality in the distribution of specialists. The Gini coefficient values for specialists ranged from 0.4 to 0.8, indicating a high to complete level of inequality. Furthermore, after the HTP, certain specialties showed a movement toward absolute inequality. Specifically, gynecologists in 2016 and 2017, psychiatrists in 2014 and 2016, neurologists in 2015 and 2017, and orthopedists in 2016 and 2017 demonstrated a shift toward absolute inequality in their distribution.

Specialized domains, such as cardiology, demonstrated a notable presence of absolute inequality, as evidenced by the Gini index before the implementation of the HTP. However, there has been limited progress in addressing this disparity through the HTP. In fact, certain specialized fields have experienced an exacerbation of inequality despite the implementation of the plan. The equitable distribution of specialists is a pressing issue that receives significant attention on a global scale, particularly in high-income countries. In the context of England, despite a relatively low doctor-to-population ratio, the distribution of doctors within the society appears to be more equitable compared to other nations. However, there are noticeable disparities in health indicators among different regions within England, resulting in certain areas having a doctor-to-population ratio that is more than twice as high as others [20]. In contrast, when comparing Yugoslavia and the USA, it has been observed that Yugoslavia has been more successful in effectively regulating the distribution of specialists compared to the USA. This can be primarily attributed to improved accessibility to healthcare workers, insurance agents, and other relevant institutions [21]. Japan is currently facing a significant healthcare challenge due to a shortage of doctors. Among the 30 member countries of the Organization for Economic Cooperation and Development (OECD), Japan ranks among the bottom four countries in terms of the doctor-to-population ratio, indicating a considerable scarcity of doctors relative to its population [22]. Australia and Canada also face similar challenges in terms of doctor distribution, struggling with the issue of ensuring an equitable distribution of doctors throughout their respective countries. [7].

A study conducted by Amini et al. investigated the dispersion of specialists and found a significant degree of dispersion in their distribution. This indicates that specialists are unevenly distributed across the studied population [7]. Similarly, in the context of Japan, despite an increase in the number of specialist staff, their distribution has remained unfavorable and unchanged [23]. Another study conducted by Qadri in 2020 revealed inequality in the distribution of specialists in the cities of Sistan and Baluchistan province. These findings are consistent with the results of previous studies, including the one mentioned above [19].

Our study findings suggest that there is a significant level of inequality in the distribution of subspecialties, as indicated by the Gini coefficient. However, there have been some positive developments in certain subspecialty fields in Qazvin province following the implementation of the HTP. Specifically, subspecialties such as allergy and immunology, perinatal medicine, pediatric oncology, and vascular and heart and kidney surgery for children have shown improvements in their distribution. This indicates a more balanced allocation of these subspecialties in the province.

When comparing the Gini coefficient values of specialized and subspecialized fields, both categories demonstrate a high level of inequality. In subspecialized fields, the Gini coefficient typically ranges from 0.4 to 0.6, indicating a significant degree of inequality. Similarly, in specialized fields, the Gini coefficient ranges from 0.4 to 0.8, representing a range from poor to complete inequality. Furthermore, a study conducted by Nouraei et al. in 2014 focused on the Gini coefficient of radiology centers. The findings revealed that the distribution of these centers became increasingly unfair over time, with the Gini coefficient increasing from 0.38 in 2006 to 0.49 in 2014. This indicates a growing level of inequality in the distribution of radiology centers [24]. Similarly, a study conducted in China examined the Gini coefficient of CT scan centers in 2006 and 2009, which yielded values of 0.14 and 0.15, respectively [25]. Likewise, a study conducted in Japan demonstrated a significant disparity in the geographical distribution of diagnostic imaging devices, which aligns with the findings of the present study [5].

According to the HHI, there is a high concentration of GPs in the cities of Qazvin province. This indicates an imbalance and inequality in the distribution of GPs relative to the population in these cities. However, it is important to note that there has been a gradual decrease in the level of inequality in the distribution of GPs over the years, except for 2016. In contrast to our findings regarding the distribution of GPs in Qazvin province, a study conducted by Qadri et al. in Sistan and Baluchistan province reported an average concentration of GPs based on the HHI. This suggests a relatively more balanced distribution of GPs in that province [19].

Based on our findings, it seems that the HHI has demonstrated diverse trends across different specialties. In the case of urology, there has been a gradual decrease in the index, which suggests a reduction in inequality in the distribution of urologists. However, it is important to note that despite this decrease, some level of inequality still exists in the distribution of urologists. In the infectious specialty, the HHI has generally remained stable, with the exception of an increase in 2017. This indicates a consistent level of inequality in the distribution of specialists in this field. On the other hand, in general surgery, the index value is low, which suggests a relatively balanced distribution of specialists. However, it is crucial to acknowledge that even with a low index value, some degree of inequality still exists in the distribution of specialists in this specialty. In the fields of anesthesia and neurology, although the HHI is low, suggesting a relatively balanced distribution of specialists, it does not imply complete equality. There may still be some degree of inequality in the distribution of specialists in these fields in the field of pathology, there was an increase in concentration observed in 2014 and 2015. This indicates a higher level of inequality. In sports medicine, concentration and inequality are still evident. It is worth noting that the findings of Qadri et al. regarding the HHI in specialists differ from our study's results. Their study reported an increase in the index from 0.2 in 2009 to 0.18 in 2017, which suggests a shift toward higher inequality in the distribution of specialists [19].

Based on the findings, it appears that there has been an increase in concentration within the fields of allergy and immunology, vascular surgery, and pediatric cardiology and urology by 2017. Furthermore, it is mentioned that high concentration levels have persisted in other specialized fields, indicating that the patterns of inequality were already established prior to the examined time period. However, it is noted that no studies examining the concentration in the distribution of subspecialists were located. This indicates a gap in the available research regarding the specific distribution of subspecialists and their concentration levels.

The concentration of doctors in provincial centers and more developed cities, which are characterized by better living conditions and higher income, can lead to an exacerbation of healthcare worker concentration, including doctors. This concentration has negative implications for the health of individuals residing in less privileged cities or regions. The findings in Qazvin province, both at the specialized and subspecialized levels, support this phenomenon. The scarcity of doctors in cities within Qazvin province has resulted in patients being redirected to provincial centers, leading to increased healthcare costs for patients. This situation contradicts the goals set forth by the HTP. The rise in costs is expected to contribute to a decline in patient satisfaction.

Our results indicate that the HTP has not been successful in achieving fair distribution of human resources. Since one of the goals of the HTP is to increase the retention of physicians in underserved areas, the values obtained from the Gini coefficient and HHI indicate concentration in the distribution of human resources. The findings show that although there has been an increase in the number of specialists at QUMS, an examination of the distribution and equality within it reveals concentration, which contradicts the implementation goals of the HTP. Based on the conducted assessments, the Gini coefficient for GPs, specialists, and subspecialists shows a significant level of inequality. In fact, simultaneous with the implementation of the HTP, the presence of specialists in the province increased, and it was expected that the distribution of these forces would be implemented with the goal of retaining physicians in underserved areas. However, due to incorrect predictions, lack of structural changes, and haste in implementing the plan, the concentration of these physicians in the central areas of the province has increased, and significant changes are not observed in underserved areas.

### Rigor of study

This study represents the first of its kind conducted in Iran, but it encountered certain limitations during its execution. One major limitation was the unavailability of accurate information regarding the number of GPs, specialists, and subspecialists. To overcome this challenge, data from multiple sources were obtained, which inadvertently extended the duration of the study beyond the initially anticipated timeframe. Additionally, it should be noted that this type of information is typically released at lengthy intervals, further adding to the complexity of data collection and analysis.

## Conclusion

The findings of this study suggest that the HTP has not effectively achieved its objective of promoting an equitable distribution of human resources in the healthcare sector. The estimated Gini coefficients for doctors in Qazvin province reveal a wide range of inequality, indicating significant disparities in the allocation of healthcare professionals. Additionally, the results obtained from the HHI highlight a concentration of doctors within the health sector of Qazvin province, which is disproportionate and potentially detrimental. This concentration could lead to a shortage of specialists in certain cities, resulting in an inadequate level of healthcare services. In order to address these challenges, it is crucial to accurately and fairly assess the healthcare workforce requirements. By doing so, it will be possible to reduce healthcare costs for families and enhance the overall efficiency of health resources. These measures are essential for ensuring equitable access to healthcare services and improving the overall quality of healthcare delivery in Qazvin province.

## Data Availability

The datasets used and/or analyzed during the current study available from the corresponding author on reasonable request. The entire dataset is in Farsi language. The data can be available in English language for the readers and make available from the corresponding author on reasonable request.
